# Quantitative Evaluation of Spatial Accessibility of Various Urban Medical Services Based on Big Data of Outpatient Appointments

**DOI:** 10.3390/ijerph20065050

**Published:** 2023-03-13

**Authors:** Jinling Sui, Guoqin Zhang, Tao Lin, Nicholas A. S. Hamm, Chunlin Li, Xian Wu, Kaiqun Hu

**Affiliations:** 1 Key Laboratory of Urban Environment and Health, Institute of Urban Environment, Chinese Academy of Sciences, Xiamen 361021, China; 2 University of Chinese Academy of Sciences, Beijing 100049, China; 3 Xiamen Key Laboratory of Smart Management on the Urban Environment, Institute of Urban Environment, Chinese Academy of Sciences, Xiamen 361021, China; 4 School of Geographical Sciences, University of Nottingham, Ningbo 315100, China; 5 CAS Key Laboratory of Forest Ecology and Management, Institute of Applied Ecology, Chinese Academy of Sciences, Shenyang 110016, China

**Keywords:** medical service spatial accessibility, 2SFCA, type of medical service, residents in different ages, big data, Xiamen City

## Abstract

Equity of urban medical services affects human health and well-being in cities and is important in building ‘just’ cities. We carried out a quantitative analysis of the spatial accessibility of medical services considering the diverse demands of people of different ages, using outpatient appointment big data and refining the two-step floating catchment area (2SFCA) method. We used the traditional 2SFCA method to evaluate the overall spatial accessibility of medical services of 504 communities in Xiamen city, considering the total population and the supply of medical resources. Approximately half the communities had good access to medical services. The communities with high accessibility were mainly on Xiamen Island, and those with low accessibility were further from the central city. The refined 2SFCA method showed a more diverse and complex spatial distribution of accessibility to medical services. Overall, 209 communities had high accessibility to internal medicine services, 133 to surgery services, 50 to gynecology and obstetrics services, and 18 to pediatric services. The traditional method may over-evaluate or under-evaluate the accessibility of different types of medical services for most communities compared with the refined evaluation method. Our study can provide more precise information on urban medical service spatial accessibility to support just city development and design.

## 1. Introduction

The pursuit of a ‘just’ city is a vital part of the construction of a sustainable and resilient city, but equity considerations are still lacking in development decisions [1,2]. A ‘just’ city can be defined as one in which everyone has an equal opportunity to thrive, and health outcomes and environmental benefits are shared equitably, regardless of people’s economic status, gender, age, ethnicity, religion and ability [3]. Historically, urban improvements in education, housing and public health have led to longer life expectancy and healthier populations. However, as infectious diseases and malnutrition have declined in many cities, noncommunicable diseases, including physical and mental health conditions, have become more prevalent [4]. “Ensure healthy lives and promote well-being for all at all ages” is the third of the seventeen sustainable development goals (SDGs) in the United Nations (UN) 2030 Agenda for Sustainable Development [5]. People of different generations and different social classes need health equity. Vulnerable groups, such as young children, older or poorer people, and those with compromised health, are at greater risk of disease. Cities need to provide well-designed interventions and policies to reduce this inequality [6]. This need has become more obvious during the COVID-19 pandemic, which has increased the demand for high-quality medical services recently [7]. COVID-19, like many other diseases, disproportionately affects vulnerable groups. Even in the most developed metropolises, rates of infection and deaths are more than twice as high among people affected by poverty. In order to combat inequalities, cities need to strengthen leadership and healthcare systems [8].

The spatial accessibility of medical services is an important topic in research on the supply–demand balance in medical services. ‘Balance’ means a spatial pattern of equity that matches the supply and demand between different study units. Spatial accessibility can be defined as the distance or time required to travel between the point of demand and the service providers. The spatial accessibility of medical services can reflect the resource distribution. Low spatial accessibility often means a shortage of medical service providers or social disparities for different areas or population groups; this should have more attention to when planning new medical service institutions. Therefore, the spatial accessibility of medical services plays a critical role in the planning and location of medical resources [9,10,11,12,13]. Scholars have developed several concepts and methods to measure the spatial accessibility of medical services [14,15,16,17], which can be classified into three categories: the ratio method, the distance method, and the gravity model. The ratio method often calculates the quantity of medical services per capita in the service areas. The distance method computes the distance from demand points to the nearest medical service provider or the average distance to a set of service providers. The gravity model is developed based on Newton’s Law of Gravitation, which measures the interaction between medical service demand points and supply locations and has great potential to evaluate spatial accessibility [12]. One commonly used gravity model is the two-step floating catchment area method (2SFCA), which integrates the demand and supply of medical services to measure the spatial accessibility of medical services and institutions in different residential locations [18,19,20,21,22].

Previous studies measuring the spatial accessibility of healthcare services have represented demand by the total population in each residential area and supply by the number of hospital beds or physicians, or the total amount of other services available [23,24,25,26,27]. However, these demand indicators did not fully consider the differences between age groups. For example, older people are higher users of medical services than young and middle-aged groups. For two communities with the same overall population but different proportions of older people, the community with more older people will probably have more demand for medical services. Therefore, more recently, many scholars have integrated the different characteristics of the potential patient population, the differences in healthcare-seeking behavior caused by population characteristics, and environmental factors into the study of factors influencing accessibility [28,29,30,31,32]. For example, some scholars have discussed the difference in medical demand intensity or the travel mode between older people and other groups [28,31,32]. Others have studied the equity of medical services associated with income levels in different communities [33], and some studies have analyzed the equality of access to public services for people of different ages under the influence of differences in demand and travel modes [34]. These studies show urban residents’ different situations in obtaining medical or public services and, therefore, better reflect the actual situation [35]. Some studies have used the “two-week prevalence” rate as an indicator [28,36]. For research purposes, this indicator is a good reflection of the number of people who need medical services. Therefore, we use this indicator, alongside the spatial distribution of the population in the study area, to explore the difference in the total number of patients caused by the age structure in different communities.

The situation is similar for supply indicators. Two hospitals with the same number of beds or physicians may use different configurations of medical services and departments. For example, some hospitals specialize in pediatric services which only serve children. Many departments can only serve specific populations. However, some previous studies have used the total number of inpatient beds to represent the supply of medical services. After obtaining the total number of potential patients in different communities, analyzing the demand of these patients for different types of medical services, and finally matching the differentiated demands from different demographic communities with different types of medical resource supply are thus necessary. Outpatient big data is a good choice for this because it is effectively the embodiment of medical service supply. Outpatient visits far outnumber inpatients stay for most medical institutions. For example, the number of outpatient visits to medical institutions in China was 7.74 billion in 2020, but there were only 0.23 billion stays. Most of the inpatients were transferred from the outpatients. The supply of outpatient resources also affects the behavior of patients, at least to some extent. For example, the type and content of outpatient resources can directly affect the patient’s choice of a medical appointment. Outpatient big data also reflect the specific demands of patients. The patient’s appointment and clinic data represent the patient’s demand for medical service types and even reflect the patient’s disease characteristics. Outpatient data may, therefore, better reflect the medical service supply of medical institutions. The use of outpatient big data is a useful supplement to research on accessibility calculation of single-use inpatient data and the data collection of medical service resource supply data and can therefore further expand previous related research. The differences in both supply and demand should therefore be considered for healthcare between different age groups when measuring the spatial accessibility of medical services.

In this paper, we propose a quantitative evaluation method of the spatial accessibility of urban medical services by refining the two-step floating catchment area (2SFCA) method. Taking Xiamen City in China as a case study area, we try to integrate the demands of different age groups and the supply of different types of medical services to carry out a quantitative evaluation of the spatial accessibility of urban medical services using outpatient appointment big data collected from the online appointment platform to represent the supply of medical services. The spatial distribution of accessibility medical services is compared between the refined and traditional methods. Therefore, the study answers two research questions: (1) How can one quantitatively evaluate the spatial accessibility of urban medical services by considering the demands of different age groups and the supply of different types of medical services? (2) What are the differences in the evaluation of accessibility between the traditional and refined versions of 2SFCA? We hope that our study will provide more precise information on improving the spatial accessibility of urban medical services and support’ just’ city development and design.

## 2. Materials and Methods

### 2.1. Study Area

**Figure 1:**
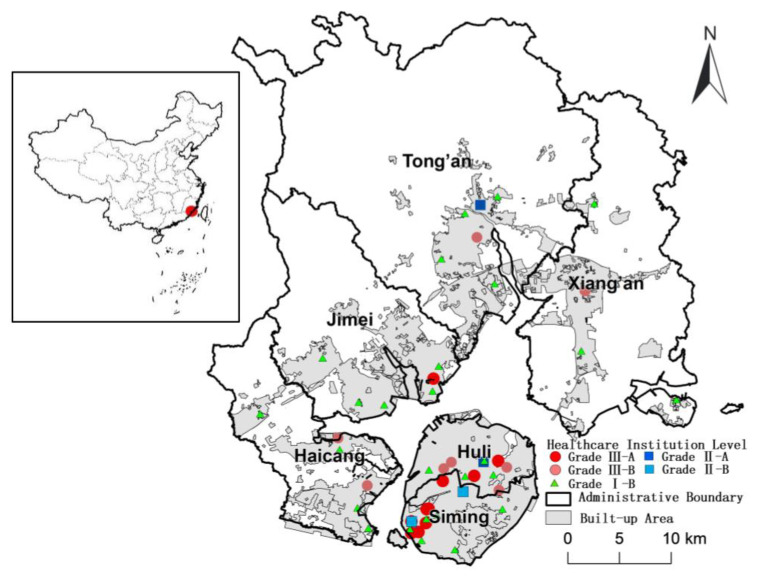
Spatial distribution of medical institutions in the six districts of Xiamen City.

Xiamen City is located on the southeastern coast of Fujian Province, China (24°23′–24°54′ N, 117°53′–118°26′ E), covering a total land area of 1700.61 km^2^. Xiamen includes the downtown area of Xiamen Island and the more suburban area of the mainland part of the city. It has six administrative districts, Siming and Huli on Xiamen Island and Haicang, Jimei, Tong’an and Xiang’an on the mainland. The city contains 51 towns and subdistricts with 504 communities. Approximately 45% of the total population is concentrated on the island, across just 157.98 km^2^ of land. As one of the four earliest Special Economic Zones in China, Xiamen has been experiencing rapid urbanization and development of the public health service since the 1980s. In the past three decades, the urbanization level of the city has increased from 35.0% in 1980 to 89.4% in 2020. The public service there has also experienced rapid development. The number of healthcare institutions increased from 336 in 1980 to 2171 in 2020, with an increase in the number of beds in healthcare institutions from 3222 in 1980 to 19,470 in 2020 and healthcare technicians from 3697 in 1980 to 38,540 in 2020. The total number of outpatient visits to hospitals and community healthcare centers was around 36 million in 2020 [37]. There are 53 public hospitals in Xiamen, of which twenty-one are tertiary hospitals (the top-grade hospitals, including 10 Level A hospitals), five secondary and twenty-seven primary (Figure 1).

### 2.2. Quantitative Evaluation Method of Urban Medical Service Spatial Accessibility

#### 2.2.1. Overall Evaluation of Urban Medical Service Spatial Accessibility

The two-step floating catchment area (2SFCA) method has been widely used to measure spatial accessibility [21,22,23,24,25]. The traditional 2SFCA method has two steps to evaluate the overall medical service spatial accessibility [16].

Step 1: For each medical service supplier location *j*, all the locations of demand (*k*) within a fixed threshold distance or travel time (*d*_0_) from supplier location *j* are searched, and the medical service demand in location *k* (*D_k_*, usually represented by population) is summed to obtain the total medical service demand (*D_j_*) for supplier location *j*. The total supply (*S_j_*, usually represented by the number of physicians) is divided by the total demand in location *j* to give the supply-to-demand ratio (*R_j_*) for supplier location *j*:

(1) $$R_{j}=\frac{S_{j}}{D_{j}}=\frac{S_{j}}{\sum_{k\in \left\{d_{kj}\leq d_{0}\right\}}D_{k}}$$

where *d_kj_* is the distance or travel time from location *k* to *j*.

Step 2: For each medical service location of demand *i*, all supplier locations (*j*) providing medical services within the threshold distance or travel time of location *j* are searched, and the supply-to-demand ratio is summed as the accessibility value of demand location *i* ($A_{i}^{F}$). A larger value indicates better accessibility of medical services from the location of demand *i*:

(2) $$A_{i}^{F}=\sum_{j\in \left\{d_{ij}\leq d_{0}\right\}}R_{j}=\sum_{j\in \left\{d_{ij}\leq d_{0}\right\}}\frac{S_{j}}{\sum_{k\in \left\{d_{kj}\leq d_{0}\right\}}D_{k}}$$

The accessibility of urban medical services calculated by the 2SFCA method reflects the balance between the supply and demand of medical services. However, the single threshold distance or travel time cannot accurately reflect the differences in service coverage and capacity of different grades of healthcare institutions. This method also does not consider the effects of travel distance or time on residents’ choice of healthcare treatment.

#### 2.2.2. Refined Evaluation of Urban Medical Service Spatial Accessibility

In this study, we refined the traditional 2SFCA method to integrate the demands of different age groups and the supply of different types of medical services. The different threshold times for different hospital grades and the effect of distance were considered. On the supply side, medical services were classified into different types by hospital departments. Only outpatient services were considered because of the availability of data. On the demand side, the total population, which has commonly been used in some studies, was divided into different groups using census data. To reflect the demand characteristics for different outpatient services, we divided the total population into four age groups: children (aged 0–14), young people (aged 15–44), middle-aged people (aged 45–64), and older people (aged ≥ 65). Different population groups’ demands for different types of medical services were calculated using a healthcare survey. To match the demand for different medical services by different age groups, we classified the medical service supply into four types by the services available at each hospital: internal medicine, surgery, obstetrics and gynecology, and pediatric services. Surgical clinics include specialized departments, such as an orthopedics department and a hepatobiliary surgery department, where surgical resection and repair are the primary means of treatment. Internal medicine clinics include digestive medicine, neurology, and other departments that generally do not carry out open surgical treatment. Obstetrics and gynecology mainly focus on female reproductive organ diseases, physiological and pathological changes of pregnancy and childbirth, and women ‘s health care. Pediatrics focuses on preventing and treating diseases that promote physiologically and psychologically healthy growth from fetus to adolescent children. The number of appointments for each type of outpatient service at each hospital was used to represent the supply size of the hospital for that service.

The total population may reflect the potential demand for medical services. However, the number of individuals with different conditions in the population provides a more accurate picture of the demand for medical services than simply the population. We used the number of patients to represent the demand size for different medical services in this study; we calculated the number of patients in each community over the relevant 2 weeks for the four types of medical services using the equation below:

(3) $$N_{is}=\left(\sum_{j=1}^{m}P_{i}\times G_{ij}\times p_{ij}\right)\times q_{is}$$

where: $N_{is}$ is the number of patients in community *i* for outpatient services, $P_{i}$ is the total population of community *i*, $G_{ij}$ is the proportion of age group *j* in community *i*, $p_{ij}$ is the prevalence of patients in the 2 weeks in age group *j* in community *i*, and $q_{is}$ is the proportion of patients who need outpatient services in community *i*.

Table 1 shows the 2-week prevalence data ($p_{ij}$) for different age groups collected from the *Fifth National Health Service Survey in China* [38]. The proportion of patients who needed certain types of outpatient services in each community was calculated using the proportion of outpatient visits for each type of outpatient service (Table 2) derived from the *China Health Statistics Yearbook* [39].

**Figure 2:**
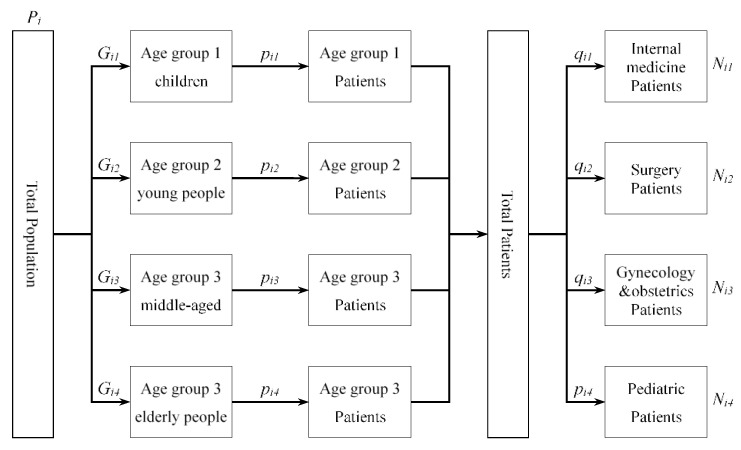
Calculation process for patients of the four types of medical services.

The calculation process for Equation (3) is shown in Figure 2.

Qualified hospitals in China can be classified into three grades—primary (the lowest grade), secondary and tertiary (the top grade)—by their ability to provide medical services. According to the level of medical service provision and hardware, every grade can also be further divided into Level A (the top level), and Level B. Hospitals in China can therefore be classified into a three-grade, six-level system, with the highest level being Tertiary Level A hospitals (Table 3).

We used the grades of hospitals to refine and calculate the 2FSCA. The method was refined and calculated as shown in Equation (4).

(4) $$A_{i}^{F}=\sum_{j\in \left\{d_{ij}\right.\left.\leq d_{0}\right\}}^{\;}\frac{S_{j}\times f{\left(d_{ij}\right)}_{1}}{\sum_{k\in \left\{d_{kj}\right.\left.\leq d_{0}\right\}}\;D_{k}\times f{\left(d_{ij}\right)}_{2}}$$

(5) $$f{\left(d_{ij}\right)}_{2}=\left\{\begin{array}{c} g\left(d_{ij}\right),\;\;\;\;\;\;\;\;\;\;\;\;d_{ij}\leq d_{0}\\ \;\;\;\;\;\;\;\;\;\;0,\;\;\;\;\;\;\;\;\;\;\;d_{ij}>d_{0} \end{array}\right.$$

(6) $$g\left(d_{ij}\right)=\frac{e^{-1/2\times {\left(d_{ij}/d_{0}\right)}^{2}}-e^{-1/2}}{1-e^{-1/2}}$$

where *f*(*d_ij_*)_2_ is the general form of the distance decay function, and *g*(*d_ij_*) is the Gaussian distance attenuation function used in this study within the search radius *d*_0_.

In the first step, we introduced the multi-level threshold distance or travel time to reflect the difference in service coverage of different grades of medical institutions (*f*(*d_ij_*)_1_). Medical institutions at different levels offer reimbursement of medical expenses to residents with medical insurance, but the characteristics and reimbursement rates of medical institutions of different levels vary. Primary hospitals are community health service centers providing preventive healthcare and basic medical services. They tend to provide services mainly for the surrounding communities. The threshold travel time (*d*_0_) of this level of healthcare institution was set to 20 min. Secondary hospitals have more departments and medical services than primary hospitals. Therefore, the threshold travel time (*d*_0_) of this level was set to 30 min. Tertiary hospitals have the most departments and healthcare resources. They have a larger number of technical professionals and better medical equipment and resources to deal with a wider variety of diseases. Some of these hospitals even provide medical services to residents from outside Xiamen City; thus, the threshold travel time at this level was not limited to the study area.

To integrate the effect of distance, some scholars have used distance decay functions such as kernel density, gravity model, or piecewise attenuation [10,40,41,42,43]. The differences between these attenuation forms are mainly reflected in the different attenuation trends. The segmentation type divides the distance attenuation function within the search radius into segments. At a closer distance, the segment has a greater weight, and the accessibility is better, which is a jump attenuation. Gravity 2SFCA and kernel density 2SFCA use the distance attenuation function of the gravity model and kernel density type as the distance attenuation function within the search radius of 2SFCA. The attenuation functions of these two power functions are both continuous, but one is convex, and one is concave. The Gaussian attenuation function is an ‘S‘ type attenuation, and the attenuation rate of accessibility with distance is slower in the near and far stages and faster in the middle part. The use of the Gaussian function is the most common. When measuring the accessibility of medical services, the choice of distance attenuation function should reflect the characteristics of patients ‘use of medical resources [43]. This study evaluates the accessibility of different types of medical services for potential patients; thus, the Gaussian function is appropriate. We used the Gaussian decay function to optimize the second step of the 2SFCA to reflect the impact of travel distance and time on residents and their choice of healthcare treatment. For residents in the same community, a lower travel time was considered to show better accessibility to medical institutions with the same service capacity.

The accessibility of each type of medical service was classified into five levels using a five-point Likert-type scale [44], where Level 1 was the lowest and Level 5 the highest, using the equal interval method. We defined Levels 1 and 2 as low, Level 3 as medium, and Levels 4 and 5 as high. To highlight the difference between these two different methods, we subtracted the accessibility levels of each community obtained using the refined method from those obtained with the traditional method and classified them into the four types from under-evaluated through to over-evaluated.

### 2.3. Data Source

**Figure 3:**
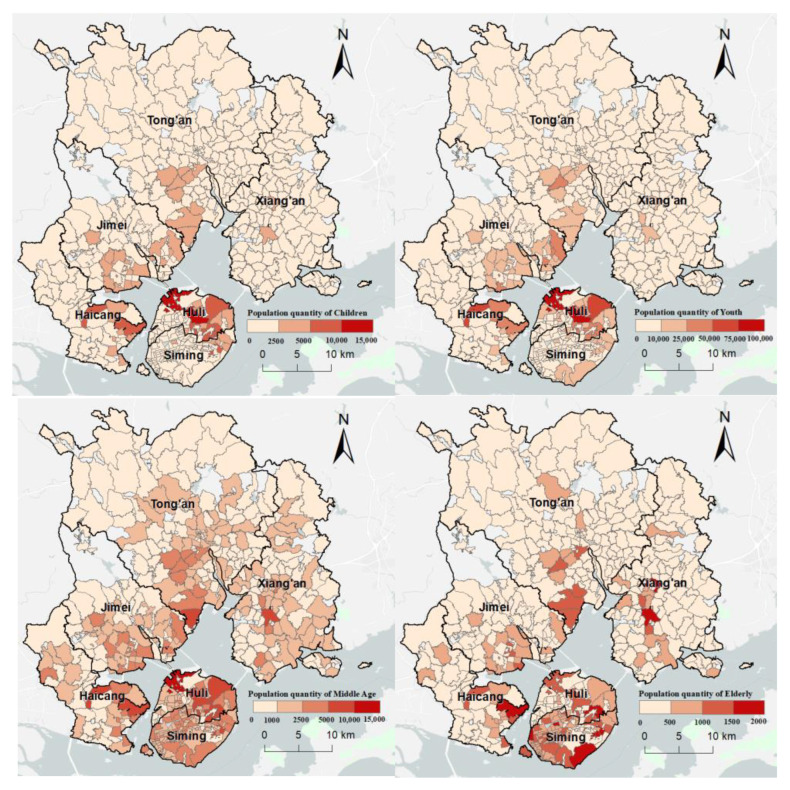
Spatial distribution of different age groups at the community scale in Xiamen City.

The data for 2 weeks of outpatient appointments (1–15 August 2018) at different hospital departments were collected from the Unified Appointment Platform for Outpatient Clinics of Xiamen City (www.xmsmjk.com/UrpOnline, accessed on 15 August 2018). The appointment information included the appointment time, doctor’s name and level, outpatient department name, hospital name, hospital address and hospital grade. We collected data on the community-scale permanent population and the age structure in 2019 from the Xiamen Municipal Bureau of Natural Resources and Planning (Figure 3).

Travel time to hospitals was computed using a route planning application programming interface, an approach that has also been used in other studies to provide more realistic travel times using real-time updated road network condition data [40]. The route planning application programming interface used here was provided by the Chinese WebGIS provider Baidu Map (lbsyun.baidu.com, accessed on 10 May 2022). Most outpatient visits occur in the daytime; thus, the computation of travel time was between 10 a.m. and 5 p.m. to eliminate the impact of morning and evening peak hours on travel time.

## 3. Results

### 3.1. Overall Accessibility of Urban Medical Service Supply-Demand in Xiamen City

**Figure 4:**
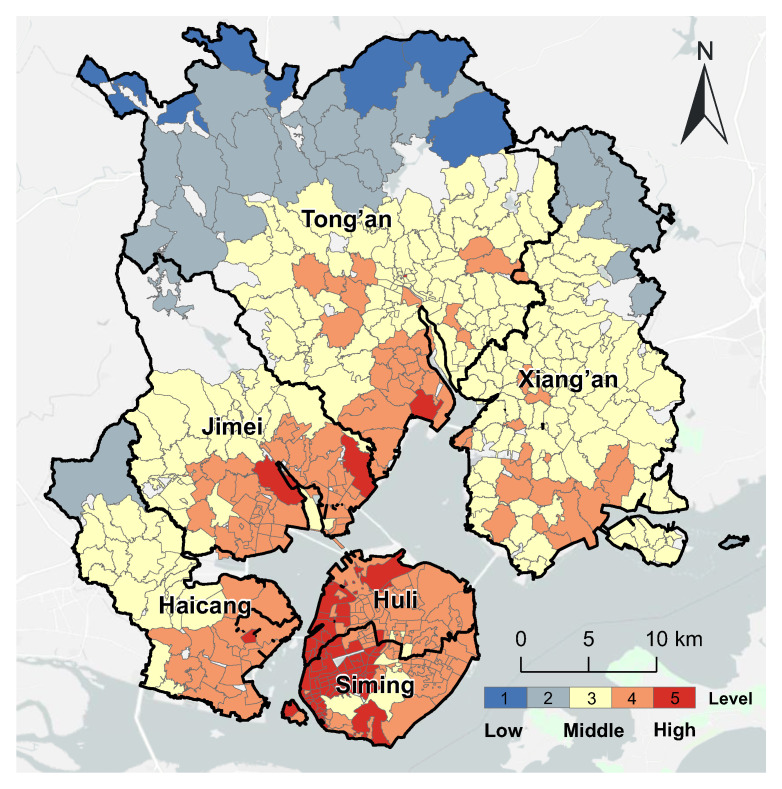
The overall accessibility of urban medical services in Xiamen City.

Using the traditional 2SFCA method, we first evaluated the spatial accessibility of medical services in each community of Xiamen city, considering the demand of the total population and supply of total medical resources. The results are shown in Figure 4. Approximately half (49.4%) of the communities had high levels of accessibility to medical services. These communities were mainly located on Xiamen Island, with some in the other four mainland districts close to the island. Most of the Level 5 communities were concentrated in the west of Xiamen Island, where there are several hospitals. The 221 communities (43.85%) with medium accessibility were in the northeast and northwest of Xiamen City. Only 34 communities (6.75%) had low accessibility and were all concentrated in the north of the Tong’an District, which is furthest from the downtown area.

### 3.2. Refined Accessibility of Urban Medical Service Supply-Demand in Xiamen City

#### 3.2.1. Spatial Accessibility of Internal Medicine Services

**Figure 5:**
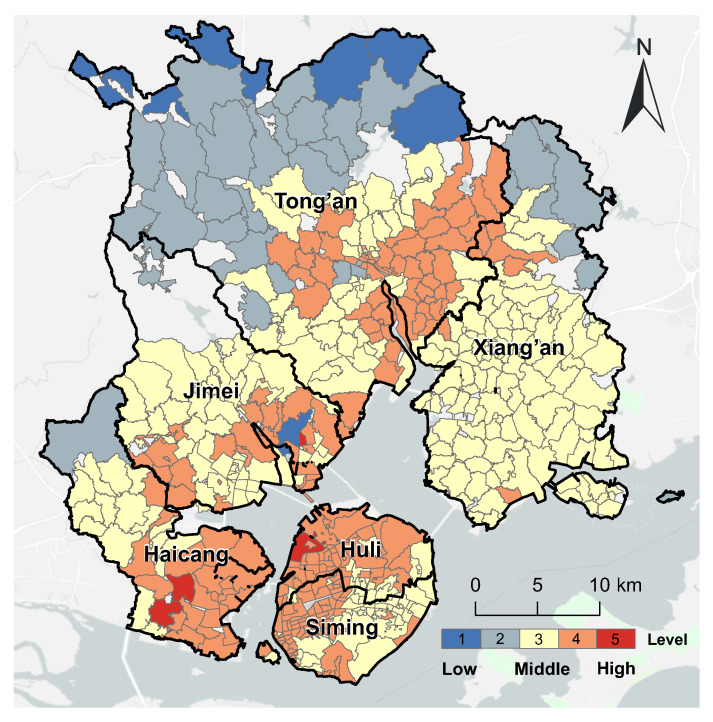
The spatial distribution of accessibility of internal medicine services in Xiamen City.

Figure 5 shows the spatial distribution of the accessibility of internal medicine services in Xiamen City. There were 209 communities with high spatial accessibility (41.47%) concentrated in the southwest and northeast of Xiamen City. The 252 communities (50.00%) with medium accessibility were mainly located in the Jimei and Xiang’an Districts. Most of the 43 communities (8.53%) with low spatial accessibility were in the north of the Tong’an District, a largely rural area far from the island. More than 90% of communities had medium or high spatial accessibility of internal medicine services, showing that there were plenty of resources for this specialty for most communities across different age groups in Xiamen City.

**Figure 6:**
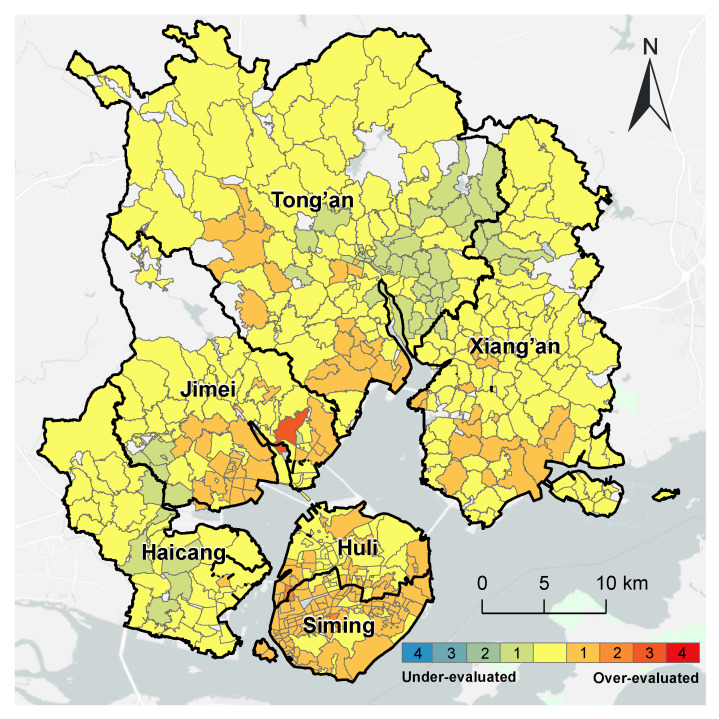
Comparison of the accessibility of internal medicine services with the overall accessibility in Xiamen City.

Compared with the refined evaluation of internal medicine services, the evaluation using the traditional 2SFCA method has over-evaluated the spatial accessibility of 167 communities (approximately one-third of the total), which were concentrated on Xiamen Island and the south of the Jimei District, Tong’an District, and Xiang’an District. Meanwhile, 53 communities (approximately one-tenth) were under-evaluated, which were in the north of the Xiang’an District and the middle of the Haicang District (Figure 6).

#### 3.2.2. Spatial Accessibility Evaluation of Surgery Services

**Figure 7:**
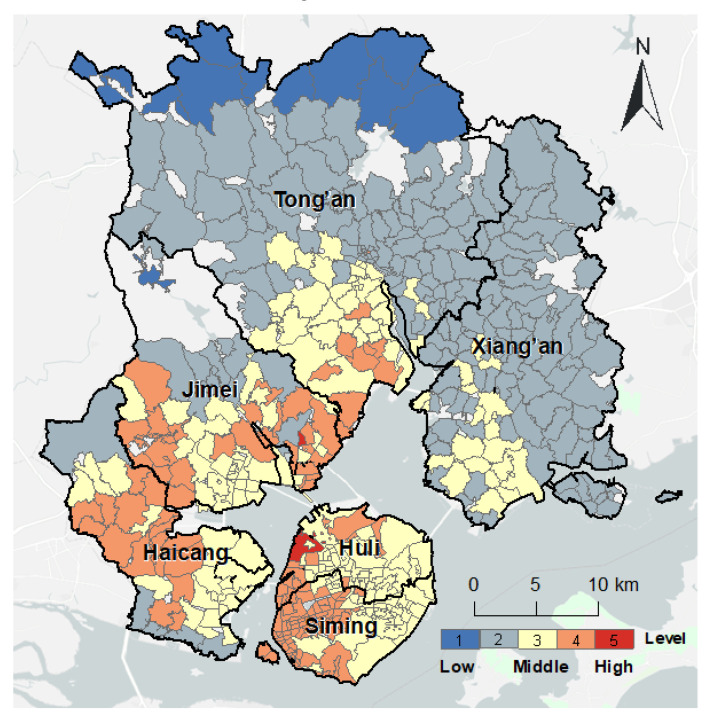
Spatial distribution of accessibility of surgery service in Xiamen City.

Figure 7 shows the spatial distribution of the accessibility of surgery services in Xiamen City. There were 133 communities (26.39% of the total) with high accessibility concentrated in the southwest of Xiamen City. The 172 communities (34.13%) with medium accessibility were mainly located in the east of Xiamen Island, east of the Haicang District and south of the Tong’an District. Most of the 199 communities (39.48%) with low accessibility were in the Tong’an and Xiang’an Districts. Overall, communities on Xiamen Island had better accessibility to surgery services than those outside the island.

**Figure 8:**
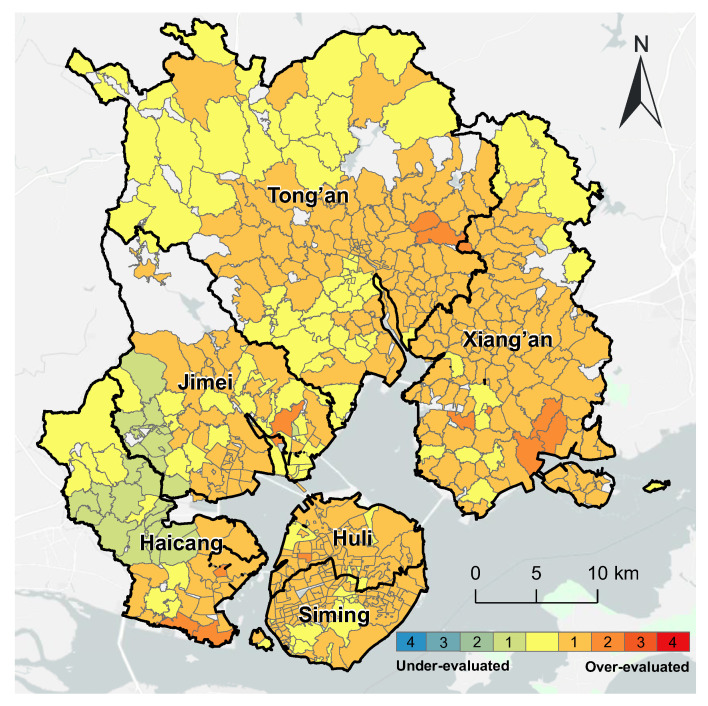
Comparison of the spatial accessibility of surgery service with the overall spatial accessibility in Xiamen City.

Compared with the refined evaluation for surgery services, the overall accessibility evaluation over-evaluated the spatial accessibility of 366 communities (more than 70% of the total), most of which were concentrated in Xiamen Island, Xiang’an District, and in the south of the Tong’an District, Jimei District, and Haicang District. A total of 24 communities were under-evaluated, all in the west of Haicang District (Figure 8).

#### 3.2.3. Spatial Accessibility Evaluation of Gynecology and Obstetrics Services

**Figure 9:**
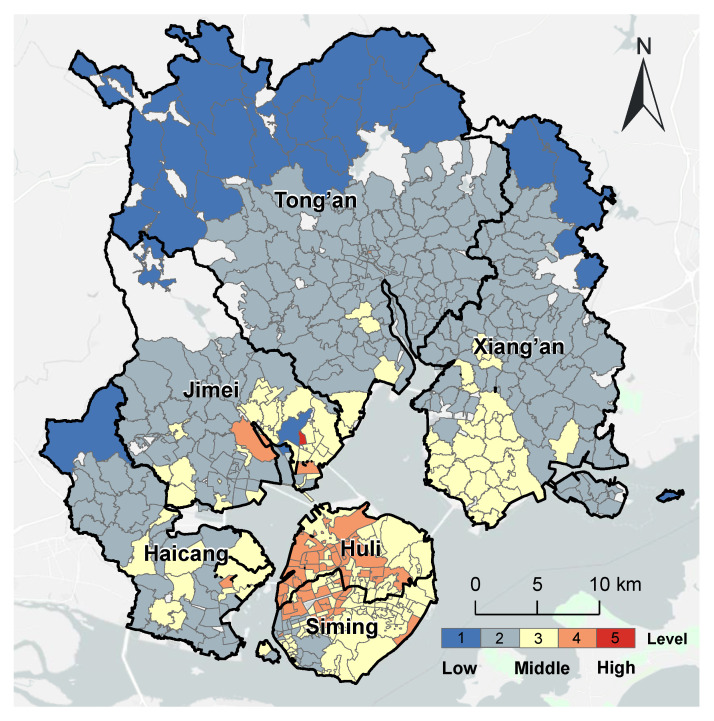
Spatial distribution of accessibility of gynecology and obstetrics services in Xiamen City.

Figure 9 shows the spatial distribution of the accessibility of gynecology and obstetrics services in Xiamen City. There were only 50 communities (9.92%) with high accessibility, and they were mostly concentrated in the northwest of Xiamen Island. Overall, 154 communities (30.56%) had medium accessibility, and they were in the southeast of Xiamen Island, the east of the Haicang and Jimei Districts and the southwest of Xiang’an District. A total of 59.52% of the communities had low accessibility. Most of these communities were outside the island, where it was harder to access gynecology and obstetrics services than on Xiamen Island.

**Figure 10:**
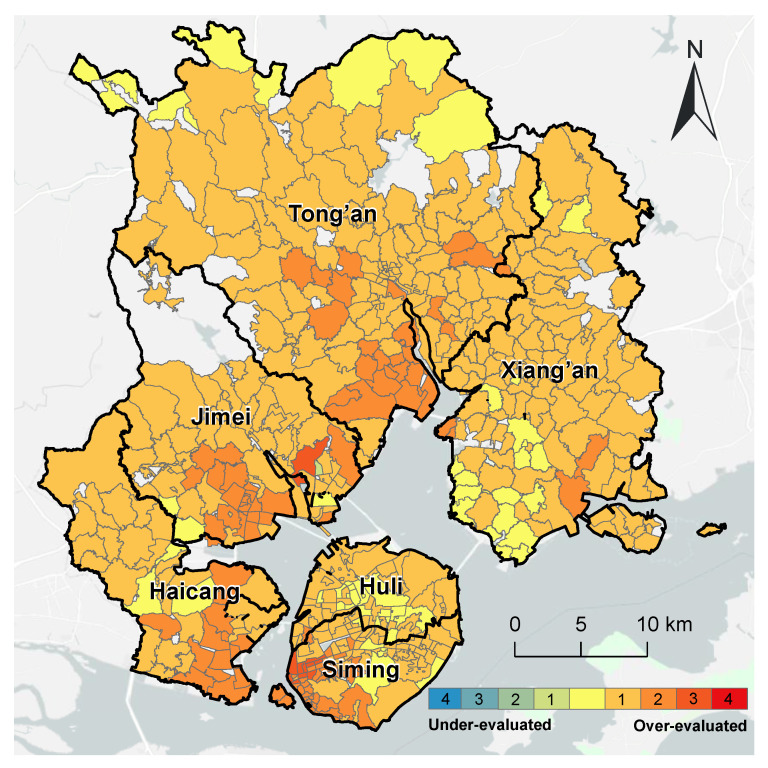
Comparison of the accessibility of gynecology and obstetrics service with the overall accessibility in Xiamen City.

Compared with the refined evaluation for gynecology and obstetrics services, the overall accessibility evaluation has over-evaluated the accessibility of 451 communities (about 90% of the total). Only 52 communities (about one-tenth) had consistent results from both methods. Those communities were distributed in the north of the Tong’an district, in the south of Xiang’an District, in the center of the Haicang District, and in the middle of Xiamen Island (Figure 10).

#### 3.2.4. Spatial Accessibility Evaluation of Pediatric Services

**Figure 11:**
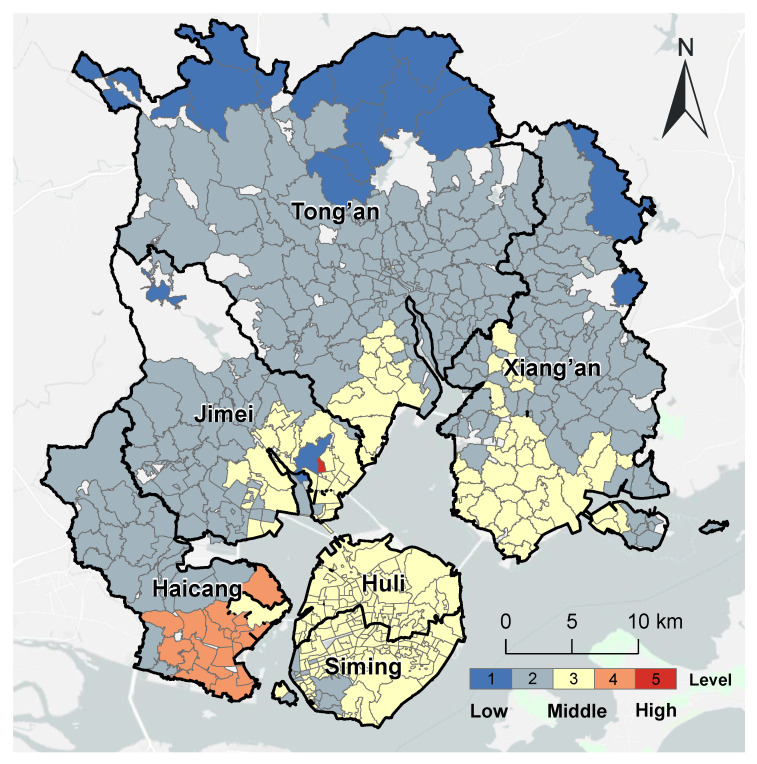
Spatial distribution of accessibility of pediatric service accessibility in Xiamen City.

Figure 11 shows the spatial distribution of the accessibility level of pediatric services in Xiamen City. This was significantly different from the other three types of medical services. In total, just 18 communities (3.57% of the total) had high accessibility to pediatric services. Only one community in the Jimei District had Level 5 accessibility to pediatric services. The 17 communities with Level 4 accessibility of pediatric services were all concentrated in the east of the Haicang District. Most of the 216 communities (42.86%) with medium accessibility to pediatric services were on Xiamen Island. More than half (53.57% of the total) of the communities had low spatial accessibility to pediatric services. These communities are concentrated in the north and west of Xiamen City.

**Figure 12:**
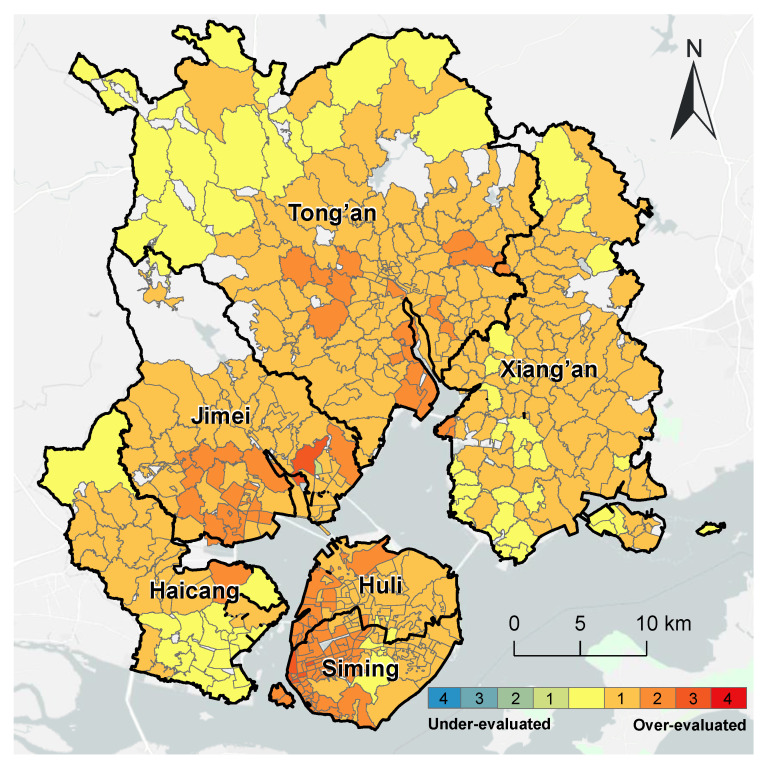
Comparison of the accessibility of pediatric services with the overall spatial accessibility in Xiamen City.

Compared with the refined evaluation for pediatric services, the overall evaluation over-evaluated the accessibility of 433 communities (approximately 85% of the total). Only 70 communities (approximately 14%) had consistent results from the two methods. They were distributed in the north of the Tong’an district, in the south of the Xiang’an District, in the south of the Haicang District, and the middle of Xiamen Island (Figure 12).

## 4. Discussion

### 4.1. Advantage of Refined Spatial Accessibility Evaluation of Urban Medical Services

In an overall accessibility evaluation using the traditional 2SFCA method, the total number of hospital beds and total population are often used to represent medical supply and demand. The uneven distribution of accessibility to medical services was only related to the spatial distribution of overall healthcare resources and population density. However, this approach does not fully consider the supply of different types of medical services and the medical needs of different population groups. Our refined spatial accessibility evaluation method can consider the age structure of the population and different types of medical services. This approach integrated population structure, the prevalence of illness among residents, and the use of outpatient departments. It can, therefore, reflect the real demands for medical services in urban communities better. It can also explore the differences in service types and capacity of medical institutions better by integrating personnel information and outpatient big data for each department of each medical institution. In summary, the refined spatial accessibility evaluation of urban medical services can accurately depict the spatial patterns of the supply–demand balance of different types of public medical services. This method can also integrate big data and other data sources. For example, the introduction of WebGIS route planning APIs (Applications Programming Interfaces), the multi-level threshold travel time, and the Gaussian decay function based on the hospital grades made our evaluation results more consistent with reality. Therefore, this refined method has great potential to provide more specific and detailed information to support better urban medical service development and design.

### 4.2. Urban Medical Service Spatial Accessibility towards Just City Construction

Equity means fairness of rights, distribution and access. Maximizing the accessibility of urban public services provides an opportunity to support ‘just’ city construction. The refined method for evaluating accessibility can support the planning and optimization of healthcare resources at the departmental or personnel level, as well as by location and grade of the hospital towards ‘just’ city construction. For example, the spatial pattern of the pediatric services was obviously different from the other three types. More pediatric services were needed for most communities in Xiamen City, except in the south of the Haicang District. In contrast, most communities with low accessibility to gynecology and obstetrics services were located mainly in the south of the Haicang District. Therefore, this area needs more of these specific medical resources in the near future. From the demand side, the acceleration of population aging, the increase in chronic diseases, and the improvement of residents‘ health awareness have increased the demand for healthcare services. On the supply side, medical resources are insufficient and unevenly distributed, making it difficult to match supply with the rapidly growing demands for healthcare. In the future, as the demographic structure changes and the supply of medical services improves, according to a certain standard, e.g., the number of doctors per person proposed in policy documents (especially in particular areas of the city) there may be an oversupply of medical services. When this happens, the oversupply of medical resources should be reflected by introducing corresponding parameters in the accessibility measures.

There are imbalances in other types of urban public services, such as education, green space, commercial services, and healthcare. Inequality exists on different scales, both within the different city regions and between both cities and countries [45,46]. In different geographic and socio-economic contexts, the promotion of fairness or justice in city construction should focus on local situations and specific problems. The refined 2SFCA method can be used in planning medical services and other urban public resources to promote the implementation of equity-based policies.

### 4.3. Limitation of the Research

In this study, only demographic differences within communities were included in the analysis of variations in demand for medical services. Future studies could consider more demographic characteristics, such as gender or income level, which may also influence the demand for different types of medical services. Second, outpatient appointments were used to represent the supply of different types of medical services. Future studies might integrate physician numbers and beds in different departments of each hospital. Third, different groups of people may use different transportation modes to access medical services. For example, differences in the characteristics of the population may lead to greater variations in travel patterns; older people are more likely to use public transport and less likely to drive than people of other ages [28], people with high incomes are more likely to be able to drive themselves to appointments [33]. The impact of travel modes on accessibility results should therefore be considered when carrying out research among different groups. Future studies might include more transportation modes, diversified search radii, and different distance attenuation decays to fit the demographic characteristics of different communities. When conditions permit, we also hope to study the impact of factors such as the accessibility of medical resources on residents’ specific healthcare-seeking behavior and then consider the health effects of healthcare-seeking behavior.

## 5. Conclusions

Previous evaluations of the overall accessibility of healthcare services using the traditional 2SFCA method often used the total number of hospital beds and total population to represent medical supply and demand. However, this approach does not fully consider the supply of different types of medical services and the medical needs of different population groups. In this study, we propose a refined accessibility evaluation method based on the traditional 2SFCA method. The refined method integrates the age structure of the population and different types of medical services. Therefore, it brings together population structure, the prevalence of illness among residents and the use of outpatient departments. The traditional 2SFCA method was used to evaluate the overall accessibility of medical services of 504 communities of Xiamen City and found that approximately half of the communities had high accessibility to healthcare services. These communities were mainly concentrated on Xiamen Island. The refined 2SFCA method found more diversified and complex spatial distribution patterns of accessibility of medical services because of the spatial distribution of different age populations, healthcare resources and transport. The traditional method may therefore over-evaluate or under-evaluate the accessibility of different types of medical services across most communities. The refined 2SFCA method better reflects the real demand for medical services in urban communities and could also be used for other urban public resources to promote the construction of ‘just’ cities.

## Figures and Tables

**Table 1:** Two-week prevalence for different age groups.

Age	Children	Youth	Middle Age	Elderly
prevalence (‰)	9.18	7.28	33.9	73.6

**Table 2:** The proportion of outpatient visits to each service type.

Outpatient Service Types	Internal Medicine	Surgery	Gynecology and Obstetrics	Pediatrics	Total
proportion (%)	47.73	30.91	10.07	11.27	100

**Table 3:** Grades classification of qualified hospitals in China.

Grade	Primary Level B	Primary Level A	Secondary Level B	Secondary Level A	Tertiary Level B	Tertiary Level A
Code	I-B	I-A	II-B	II-A	III-B	III-A

## Data Availability

The data and models generated or used during the study appears in the submitted article.
